# Case Report: Multidisciplinary management of a patient with indolent systemic mastocytosis and refractory symptoms

**DOI:** 10.3389/falgy.2024.1401187

**Published:** 2024-10-18

**Authors:** Matthew J. Hamilton, Loren W. Greene, Lauren M. Madigan, Sa A. Wang, Cecilia Arana Yi, Andrew Kuykendall, Tracy I. George, Mariana C. Castells

**Affiliations:** ^1^ Division of Gastroenterology, Hepatology, and Endoscopy, Brigham and Women’s Hospital and Harvard Medical School, Boston, MA, United States; ^2^ Department of Medicine, NYU Grossman School of Medicine, New York, NY, United States; ^3^ Department of Dermatology, University of Utah, Salt Lake City, UT, United States; ^4^ Division of Pathology-Lab Medicine Division, Department of Hematopathology, MD Anderson Cancer Center, Houston, TX, United States; ^5^ Division of Hematology and Medical Oncology, Mayo Clinic, Phoenix, AZ, United States; ^6^ Department of Malignant Hematology, H. Lee Moffitt Cancer Center, Tampa, FL, United States; ^7^ ARUP Laboratories, Department of Pathology, University of Utah School of Medicine, Salt Lake City, UT, United States; ^8^ Department of Medicine, Brigham and Women’s Hospital and Harvard Medical School, Boston, MA, United States

**Keywords:** anaphylaxis, tryptase, tyrosine kinase, *KIT* mutation, case report, indolent systemic mastocytosis

## Abstract

Systemic mastocytosis (SM) is a rare hematologic condition characterized by the proliferation and accumulation in tissue of clonal mast cells in multiple organ systems. The release of mast cell mediators in the indolent disease type and the predominant mast cell infiltration of tissues in advanced disease contribute to the heterogeneous clinical presentation. The disease driver in >90% of adult cases is an activating *KIT* mutation, with D816V being the most frequent. Here we describe a case of a young adult male presenting with osteoporosis with associated symptoms of reflux and a history of bee sting anaphylaxis. A multidisciplinary approach to the diagnosis and management was required to minimize morbidities and prevent complications. Current best supportive care was inadequate to control the patient's disease, and a selective KIT D816V inhibitor (avapritinib) was initiated. Conventional, and advanced therapies, including those in the treatment pipeline for SM are discussed.

## Introduction

Systemic mastocytosis (SM) is a rare hematologic disorder characterized by clonal proliferation and accumulation of mast cells (MC) in different organ systems, including bone marrow (BM), skin, gastrointestinal (GI) tract, liver, spleen, bone, and lymph nodes, resulting in a heterogeneous clinical presentation (1–14) (Table 1). Adult patients can present with or without cutaneous MC lesions and systemic symptoms, often necessitating consultation with a variety of medical specialists. Approximately half of patients with SM will experience systemic MC activation leading to anaphylaxis and need for emergency care.

**Table 1:** Symptom presentation and clinical and laboratory evaluation of systemic mastocytosis (3–14).

Symptoms	Clinical evaluation	Diagnostic evaluation
Cardiovascular • Anaphylaxis with hypotension and syncope • Dizziness • Palpitations	• Complete medical history • Full body skin exam • Evaluation of symptoms • Documentation of triggers • Discussion of anaphylaxis history • Assessment of organomegaly • Evaluation of ascites	• CBC with differential • Liver function tests, including serum albumin, serum LDH, and serum ALP • DEXA bone density scan • Serum tryptase • Consideration of skin biopsy • Bone marrow or other extracutaneous organ biopsy ○ Tryptase ○ CD117, CD25, CD30, and/or CD2 • Molecular testing ○ High-sensitivity *c-KIT* D816V (e.g., PCR) and other *KIT* exon mutations ○ Myeloid mutation panel for possible additional mutations ○ FIP1L1-PDGFRA fusion if eosinophilia present
Gastrointestinal • Abdominal pain or cramping • Diarrhea • Heartburn or reflux • Nausea and/or vomiting • Gastroesophageal reflux		
Musculoskeletal • Bone pain • Muscle pain • Osteoporosis/osteopenia/osteosclerosis • Osteochondroma		
Neuropsychiatric • Depression • Anxiety • Brain fog • Lack of focus • Memory loss • Migraines		
Systemic • Anaphylaxis • Fatigue • Weight loss		
Skin • Darier's sign • Flushing • Pruritis		
Respiratory • Dyspnea • Nasal congestion • Wheezing • Throat swelling		

ALP, alkaline phosphatase; CBC, complete blood count; CD, cluster of differentiation; LDH, lactate dehydrogenase; DEXA, dual-energy X-ray absorptiometry; FIP1L1-PDGFRA, factor interacting with PAPOLA and CPSF1-platelet-derived growth factor receptor-α.

Release of MC mediators leads to organ- and tissue-specific symptoms that can be episodic or chronic, with severe exacerbations, and result in poor quality of life. Misdiagnosis and underdiagnosis occur frequently due to the multi-organ involvement and range of systemic symptoms (8, 15–23). Eighty percent of all cases of SM are indolent, and the clinical case presented focuses on challenging diagnostic and treatment considerations (Table 1) (3–14). indolent systemic mastocytosis (ISM) may present with a range of signs and symptoms, including life-threatening acute episodes of mast cell activation (24).

## Case description

A 44-year-old male truck driver slipped and fell on ice, landing on his back while at work. Over the next month, he developed lower back and calf muscle pain exacerbated by walking and standing. He sustained a lumbar spinal fracture 10 years prior during heavy lifting. The patient also reported reflux symptoms, right-sided abdominal pain, and generalized abdominal cramping with loose stools triggered by certain foods, heat, stress, and occasional alcohol use. He was regularly taking a proton-pump inhibitor (PPI) antacid for reflux and was not taking NSAIDs or narcotics.

The patient was initially seen at a spine clinic. Workup was unremarkable, but a second opinion with a neurologist for continued back pain revealed a vertebral body T3 “burst” compression fracture on MRI. Bone density exam showed osteoporosis in the spine (L1-L4 *Z* score −2.3, *T* score −2.5) and osteopenia in the hip (*Z* score −2.1, *T* score −2.4) and femoral head (*Z* score −1.2, *T* score −1.8), and bone imaging showed no evidence of osteolytic lesions. Rheumatologic workup revealed low serum vitamin D (16 ng/ml; normal range, 20–80 ng/ml) and normal thyroid, parathyroid, testosterone, and serum protein electrophoresis. Endocrinology was consulted to assess the diagnosis of osteoporosis in a young male. Laboratory testing revealed a normal celiac disease panel, normal collagen telopeptide for bone turnover (149 pg/ml, normal range 93–630 pg/ml), and an elevated serum tryptase (41.9 ng/ml; normal, <11.4 ng/ml), a minor criterion for SM. Teriparatide was prescribed for osteoporosis because of concern for exacerbation of his acid reflux with bisphosphonates.

The patient was referred to a mastocytosis center due to osteoporosis and elevated baseline serum tryptase (BST). Additional history revealed that he had anaphylaxis associated with loss of consciousness after two separate hornet sting episodes (at age 19 and 39). During the second sting, the patient required three doses of epinephrine for resuscitation. He had an additional episode of syncope when working outside on a hot day, wherein he felt the onset of flushing and dizziness before losing consciousness. He also described intermittent itching sensations on his chest and flushing associated with exercise, heat, and mental stress. In addition to back pain, he was most limited at this time by profound mental fog, with memory and concentration difficulties, finding it difficult to carry out routine tasks. He had a history of recurrent hives and allergic rhinitis.

On physical examination, he was alert and oriented and appeared healthy, with a blood pressure of 139/86 mmHg, heart rate of 81 bpm, and a body mass index of 28. Oropharynx and tympanic membranes were clear without lesions, and nostrils were mildly congested with pale and boggy mucosa with clear secretions and no polyps. Neck, heart, lungs, joints, and abdomen findings were normal. Skin was without hives, flushing, or lesions of cutaneous mastocytosis (i.e., urticaria pigmentosa). He had dermatographism with wheal and flare reactions upon skin stroking. Neurologic exam was grossly normal.

Further testing showed normal blood counts without eosinophilia (0.07 K/μl) and normal liver and kidney function tests. Abdominal CT scan revealed cholelithiasis, nonobstructing renal calculi in the left kidney, and “haziness” of the mesentery in the left upper quadrant with associated lymphadenopathy (largest lymph node, 16 × 13 mm). Upper endoscopy revealed no evidence of erosive esophagitis, Barrett's esophagus, or peptic ulcer disease. Blood tests showed elevated immunoglobulin E (IgE) level of 234 kU/L (upper limit, 100 kU/L) and negative serum-specific IgE against Hymenoptera venom antigens (test performed 5 years after last venom-induced anaphylaxis). Intradermal skin testing was positive to white-faced hornet and bee venoms. Repeat BST was 38.5 ng/ml, and 24-hour urine collection revealed elevated prostaglandin F2-α at 14,858 pg/mg (normal, <5,205 pg/mg) and N-methylhistamine at 1,905 mg/dl (normal, <1,800 mg/dl). He tested positive for *KIT* D816V mutation in peripheral blood (low level detected by droplet digital PCR (ddPCR), fractional abundance 0.08%). Due to the elevated prostaglandin and history of flushing, he began full-strength aspirin daily and was educated on the use of epinephrine. He was also prescribed daily oral cromolyn sodium and a nonsedating H1 antihistamine.

**Figure 1:**
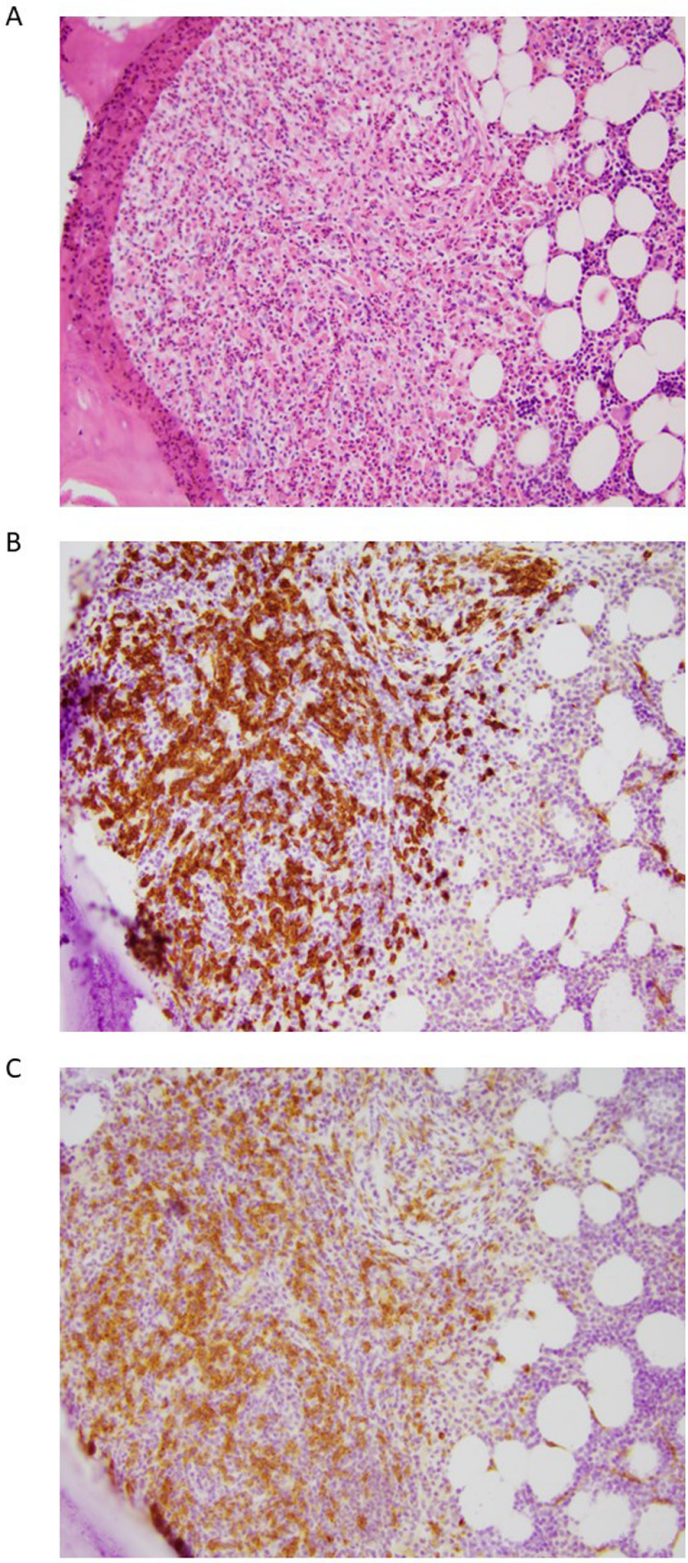
Representative histology images from a bone marrow biopsy of a patient with indolent systemic mastocytosis. The images show a mast cell aggregate in the paratrabecular space and many surrounding eosinophils. **(A)** Hematoxylin and eosin staining, **(B)** CD117 staining, **(C)** CD25 staining. All images presented at 20 × magnification.

Bone marrow biopsy revealed a moderately hypocellular marrow (30% cellular), with approximately 5% of the cellularity composed of dense MC aggregates (>15 MC) with CD25-positive MC and spindled morphology in >25% of the MC population (Figure 1). Bone marrow aspirate demonstrated that 2% to 3% of cells were MC with elongated nuclei and abundant cytoplasm containing granules with cytoplasmic extensions and no other abnormalities. Genomic DNA extracted from unfractionated BM cells analyzed by ddPCR was *KIT* D816V-positive with a low-level allele fraction of 1.8%. BST at the time of BM biopsy was 40 ng/ml, and prostaglandin metabolites were 4,822 pg/mg (normal, <5,205 pg/mg) on 325 mg of aspirin daily. The patient met major and minor WHO criteria for ISM diagnosis; B or C findings, required for AdvSM diagnosis, were absent (Supplementary Table S1) (24, 25). He started daily oral compounded ketotifen and continued daily H1 antihistamines. Oral cromolyn sodium was discontinued due to equivocal efficacy and possible side effects of fatigue. Despite not having an anaphylactic event in 5 years, he was considered high risk and was therefore started on omalizumab. He additionally underwent ultra-rush desensitization immunotherapy to Hymenoptera venom, with the plan to continue immunotherapy to bee and white-faced hornet venoms.

Surgical consult determined that the abdominal lymph nodes were secondary to unrelated sclerosing mesenteritis, observed to be stable on serial imaging studies. Psychiatry was consulted for anxiety and depression, and he was started in mirtazapine and escitalopram. In follow-up with endocrinology, teriparatide was discontinued due to possible aggravation of joint pain and other side effects. Despite the original blood collagen telopeptide indicating relatively normal bone resorption, a single dose of zoledronic acid was prescribed (26). Follow-up bone density scan showed improvement in both hip (*Z* score −1.8) and spine (*Z* score −2.1).

The patient was able to return to work nearly 2 years following the vertebral fracture but continued exhibiting severe MC mediator symptoms, including flushing and mental fog, and remained limited due to ongoing mid-upper back pain attributed to paraspinal muscle spasms. It was therefore recommended that he enroll in a tyrosine kinase inhibitor clinical trial for symptomatic patients with ISM. He and his wife elected to defer the clinical trial due to their desire to start a family. He and his wife were successful in having a child, and while enjoying fatherhood, he continued experiencing ongoing symptoms of brain fog, fatigue, abdominal pain, and bone pain despite his medical regimen directed at MC mediators, including omalizumab. The patient elected to start a clinical trial with a KIT D816V-targeting tyrosine kinase inhibitor (avapritinib). To date, he has received 36 months of treatment and has noted improvement in many daily symptoms, with only occasional brain fog, fatigue, and bone pain persisting. He is no longer limited with his activities of daily living and can work full-time while parenting. His most recent BST was 7.1 ng/ml, and repeat BM biopsy showed low-level involvement with SM.

## Discussion

The World Health Organization (WHO) defines 6 SM subtypes (Supplementary Table S1) (24, 25). Bone marrow–systemic mastocytosis (BMM), indolent SM (ISM), and smoldering SM (SSM) are chronic diseases with normal life expectancy, whereas aggressive SM (ASM), SM with an associated hematologic neoplasm (SM-AHN), and MC leukemia (MCL) are considered advanced diseases and have a reduced life expectancy that varies according to subtype. Across all types, the disease driver in >90% of adult cases is an activating *KIT* mutation, with D816V being the most frequent. The 2022 international consensus classification of SM was recently updated (27).

KIT, a tyrosine kinase surface receptor expressed by all MC, is involved in MC growth, proliferation, and activation. *KIT* mutations are acquired in SM, resulting in autophosphorylation and constitutive receptor activation independent of ligand binding. These lead to uncontrolled activation and accumulation of MC in the skin, BM, and other organs, which is a histologic hallmark of SM.

Osteoporosis in a 44-year-old male is unusual. SM can be associated with osteopenia, osteoporosis, and fracture and may be missed in a screening diagnostic workup if tryptase is not measured (27, 28). The trabecular bone of the spine is the most common location of ISM-associated bone disease, possibly because the trabecular bone's higher metabolic activity is suitable for abnormal MC proliferation. Notably, the patient's intake of PPI, which decreases stomach acid and impairs calcium absorption, further increases his osteoporosis risk.

Because this patient's collagen telopeptide was not elevated, an antiresorptive drug was chosen. Oral bisphosphonates (e.g., alendronate) often are poorly tolerated by patients with a history of acid reflux, which was a significant symptom for this patient (12, 29). Bone loss in SM may occur through the receptor activator of nuclear factor κ-B ligand (RANKL) system; therefore, denosumab is theoretically preferable to intravenous bisphosphonates based on mechanism and quick onset of action (26, 28). Other osteoporosis therapies include interferon-α combined with pamidronate, which was used in a small series of patients with ISM and improved bone density (30). Romosozumab, a drug that increases bone formation and decreases bone resorption, could also be used to treat osteoporosis in patients with ISM (31).

Up to 60% of patients with SM experience GI symptoms, although the prevalence varies in published reports (32). The effect of MC mediators most specific to patients with ISM (33, 34) include intermittent nausea, abdominal cramping, and loose stools, often triggered by a food or other exposure and commonly associated with flushing and pruritus. Involvement of acid-producing receptors in the stomach may result in peptic-type symptoms and an increased risk for peptic ulcer disease. Infiltration of abnormal MC underneath the epithelial surface of the intestine can cause chronic diarrhea and malabsorption symptoms, which is typically only observed in patients with advanced SM (AdvSM).

Endoscopy and colonoscopy with biopsy are important tools to establish a diagnosis of ISM in patients with GI symptoms who have not yet had a bone marrow biopsy, or to evaluate persistent or atypical GI symptoms in patients with an established diagnosis. Endoscopic findings in indolent disease can be normal, whereas patchy or diffusely congested intestinal mucosa appears with AdvSM. Random and targeted biopsies taken throughout the colon can reveal a patchy distribution of clonal MC by hematoxylin and eosin staining, *KIT* staining, and CD25 staining (35). Sheets and aggregates of clonal MC most often are distributed below the epithelial surface (36). Aggregates of ≥15 MC is a major WHO criterion for SM, and CD25-positive staining of MC within the aggregates is a minor criterion. Management may include PPIs for peptic ulcer disease and corticosteroids, such as oral budesonide, for chronic diarrhea and malabsorption secondary to MC infiltration in AdvSM. Sodium cromolyn has demonstrated anecdotal benefit in many patients; however, there are limited published studies demonstrating its efficacy (37).

A subset of patients with ISM may present initially with anaphylaxis with severe hypotension triggered by a Hymenoptera sting without classical cutaneous urticaria pigmentosa lesions (38) and may benefit from lifelong Hymenoptera venom immunotherapy (39). Skin testing should be done 4–6 weeks after the anaphylactic event since natural desensitization could lead to false negative results. In patients with positive skin test and/or positive specific IgE, “ultra-rush desensitization” is a novel and safe modality accomplished in one day with or without addition of omalizumab (40). Lifelong Hymenoptera venom immunotherapy is recommended, as several deaths have been reported from Hymenoptera stings in allergic ISM patients who discontinued immunotherapy (41). Omalizumab given concurrently with venom immunotherapy should also be considered because cases of anaphylaxis after a sting have occurred despite immunotherapy (42). Duration of treatment with omalizumab for patients on concomitant TKI has not been studied. The patient in this case was continued on omalizumab for 12 months to account for time to maximal clinical benefit of avapritinib and to ensure that there were no further anaphylactic events.

Serum tryptase should be evaluated in patients presenting with hypotension after a Hymenoptera sting. Two values should be obtained—one at the time of the reaction and one at least 24 h later which is considered baseline (BST). If the BST is elevated (>20 ng/ml), a BM biopsy is recommended to confirm the diagnosis of SM. If the BST is <20 ng/ml but >11.4 ng/ml, peripheral blood should be tested for the *KIT* D816V mutation, and a BM biopsy is recommended if positive. Allele burden for *KIT* D816V has prognostic value, with <2% allele burden present in patients with nonadvanced disease, whereas SSM and AdvSM present with >5% and >9% allele burden, respectively (43). In patients with a hypotensive episode after a Hymenoptera sting and BST >11.4 ng/ml and <20 ng/ml, a BM biopsy is recommended in the presence of negative *KIT* D816V mutation; lower sensitivity of the peripheral blood *KIT* D816V mutation is observed in patients with low mast cell burden.

All patients with a BST >8 ng/ml and signs and symptoms that suggest mast cell involvement should be evaluated for hereditary *α*-tryptasemia (HaT), a genetic trait present in 4% to 6% of the general population and defined by expression of extra copies of the MC tryptase gene *TPSAB1* (44). An HaT diagnosis does not exclude SM, and patients with a BST >20 ng/ml are candidates for BM biopsy to rule out SM. Furthermore, because the risk of venom-induced anaphylaxis is increased in patients with ISM with coexisting HaT, it is recommended to test for *KIT* D816V if the baseline tryptase is >8 ng/ml and to perform BM biopsy if positive (45).

Cutaneous manifestations occur in the majority of adults with SM and are particularly common in the setting of ISM. Within a European registry of 1,510 adult patients, 79% presented with skin involvement (46). Typical lesions include diffuse red-brown macules and papules, 2–3 mm in diameter and monomorphic. A positive Darier's sign—erythema and wheal formation following mechanical stimulation—is often noted. In a minority of patients, telangiectatic lesions or fixed red macules may also affect the trunk and proximal extremities, although this is no longer favored as a distinct variant (1). Cutaneous manifestations in SM can be subtle or atypical (with either an uncharacteristic distribution (47–51) or morphology (52, 53)), necessitating a high index of suspicion in patients with additional compatible symptoms/signs. Patients may also present with nonspecific features, including isolated flushing, pruritus, and urticaria. Accurate identification of cutaneous lesions is essential given the implications of SM in the skin, including a high likelihood of systemic involvement in the adult population (1, 46, 54–56). Dermatology referral should be considered for appropriate characterization of disease, potential biopsy (to be interpreted by a pathologist/dermatopathologist familiar with the cutaneous histopathology of mastocytosis), and consideration of additional skin-directed therapies (57). Evolving evidence suggesting an increased risk of keratinocyte carcinoma and cutaneous melanoma in adult patients with SM further supports the importance of yearly skin surveillance (58, 59).

ISM treatment focuses on prevention and control of anaphylaxis and MC mediator–related symptoms, and treatment and prevention of osteoporosis. Available anti-mediator therapies include H1 and H2 histamine receptor blockers, leukotriene antagonists, aspirin, cromolyn sodium, and anti-IgE monoclonal antibody treatment (60, 61) (Table 2). To decrease disease burden in advanced diseases, cladribine, interferon-α, and hydroxyurea have shown efficacy. Tyrosine kinase inhibitors that target *KIT* include midostaurin and avapritinib, which were first approved for AdvSM (62, 63). In May 2023, the US Food and Drug Administration approved avapritinib for the treatment of adult patients with ISM (64, 65). Avapritinib is a selective KIT D816V inhibitor. The phase 2 PIONEER study (NCT03731260) evaluated the safety and efficacy of a low dose of avapritinib to reduce ISM symptoms and MC burden. This study met its primary endpoint—a significant reduction in total symptom scores at week 24 compared with placebo. Secondary endpoints included improved quality of life and decreased MC burden (66, 67). Midostaurin targets both *KIT* D816Y and D816V, and a phase 2 study (NCT01920204) in ISM demonstrated improvement in SM symptoms in 75% of patients at 12 weeks, 29% improvement in quality of life at 24 weeks, and reduction in MC burden (68).

**Table 2:** Treatment options for systemic mastocytosis.

Symptoms/therapy	ISM and AdvSM treatment	ISM-only treatment	AdvSM-only treatment
MC mediator and other SM-specific symptoms	Antihistamines, oral cromolyn sodium, antileukotriene, ketotifen, PPI		
Prevention of complications	Anaphylaxis—omalizumab, desensitization Osteoporosis—bisphosphonates, anti-RANKL		
Refractory symptoms (investigational)		Avapritinib, (bezuclastinib, elenestinib)	
Conventional therapy to reduce clonal MC			Cladribine, interferon-α, hydroxyurea
Targeted tyrosine kinase therapy	*KIT* D816V present—avapritinib		*KIT* D816V present—midostaurin Mutation absent or well-differentiated SM—imatinib
Investigational targeted therapy	Bezuclastinib, elenestinib	Masitinib, TL-895	

AdvSM, advanced systemic mastocytosis; ISM, indolent systemic mastocytosis; MC, mast cell; PPI, proton pump inhibitor; RANKL, receptor activator of nuclear factor κ-B ligand; SM, systemic mastocytosis.

Other tyrosine kinase inhibitors under investigation include elenestinib, a selective KIT D816V inhibitor with minimal central nervous system penetration; bezuclastinib, which targets *KIT* D816V and exon 17/18 loop mutations; masitinib, a selective inhibitor of wild-type KIT; and TL-895, a selective second-generation inhibitor of Bruton's tyrosine kinase. The safety and efficacy of each agent are currently being evaluated in ISM (elenestinib: HARBOR, NCT04910685; bezuclastinib: SUMMIT, NCT05186753; masitinib: NCT04333108; TL-895: NCT04655118) and AdvSM (elenestinib: AZURE, NCT05609942; bezuclastinib: APEX, NCT04996875).

ISM management and treatment are highly individualized and often require a multidisciplinary approach. An overall summary of this patient's case is presented in Table 3. Although symptoms can be effectively managed, some patients will have more refractory symptoms and complications, including life-threatening anaphylaxis and osteoporosis with fractures. Targeted, selective KIT D816V inhibitors have been shown to improve symptoms and survival with AdvSM and hold promise for patients with refractory non-AdvSM, including ISM. While studies support the use of current available therapies for ISM including TKIs, omalizumab, and venom immunotherapy with respect to safety and efficacy, the overall management is evolving, and additional considerations for treatment include cost and availability. Education and awareness for medical providers who treat these patients in collaboration with mast cell disorder centers are essential for proper diagnosis and management.

**Table 3:** Case summary.

Problem/symptom	Intervention/test	Specialist involved	Treatment/outcome
Osteoporosis	Bone density, baseline serum tryptase test	Endocrinologist	Bisphosphonates
Hymenoptera allergy	Serum Hymenoptera venom-specific IgE skin testing	Allergist/Immunologist	Specific allergen immunotherapy desensitization
MC mediator symptoms	24-hour urine test for MC mediator metabolites N-methylhistamine and 11β-prostaglandin F2α	Allergist/Immunologist	Medications to target MC mediators (e.g., antihistamines, cromolyn)
Anaphylaxis	Education about characteristic symptoms and proper use of epinephrine	Allergist/Immunologist	Epinephrine prescribed to all patients with SM, consider omalizumab
Cutaneous lesions	Skin biopsy	Dermatologist Dermatopathologist	Antihistamines, topical corticosteroids, phototherapy, omalizumab
Depression	Assess for commonly comorbid anxiety and depression	Psychiatrist	Medical therapies, counseling
Gastroesophageal reflux disease, peptic ulcer disease	Recognize high incidence, consider endoscopy to assess for erosive disease	Gastroenterologist	Endoscopy, use of proton pump inhibitors—caution with chronic use in patients with osteopenia/osteoporosis
Hepatosplenomegaly, lymphadenopathy	Cross-section abdominal imaging	Gastroenterologist, Allergist/Immunologist, Hematologist	Document presence to characterize mastocytosis (B-findings)
Diagnosis of systemic mastocytosis	Bone marrow biopsy	Pathologist with experience in assessment of SM	Use of appropriate immunostaining for tryptase, CD25, and CD30, flow cytometry, test for *KIT* D816V and other associated mutations

IgE, immunoglobulin E; MC, mast cell; SM, systemic mastocytosis.

## Patient perspective

It has been quite a journey to get to where I am today! Just the process of calling this mastocytosis was a challenge, especially when I was in a lot of pain. I had to tell my story and answer so many questions and do so many tests for what felt like years before I was on the right treatments. I was having so many other symptoms too, like brain fog, tired all the time, stomach pains that were not always treated. When we started the trial drug for mastocytosis I was nervous about side effects but have been thankful for it ever since. Things are not perfect, but I feel like I am getting my life back, which is a great thing.

## Data Availability

The original contributions presented in the study are included in the article/Supplementary Material. Further inquiries can be directed to the corresponding author.
