# Supplementary material for: Case Report: Multidisciplinary management of a patient with indolent systemic mastocytosis and refractory symptoms
Source: Front Allergy. 2024 Oct 18;5:1401187. doi: 10.3389/falgy.2024.1401187 (PMC11527781; doi:10.3389/falgy.2024.1401187)
Supplement: Supplementary file 1 [file Table1.docx]

Supplementary Material

# Supplementary Figures and Tables

***Supplementary Table 1.*** World Health Organization 2022 Systemic Mastocytosis Classifications^24,25^

| **Diagnosis of Systemic Mastocytosis** | | |
| --- | --- | --- |
| **Major Criteria** | **Minor Criteria** | **Diagnostic Requirements** |
| Multifocal dense infiltrates of MC (>15 MC in aggregates) in bone marrow biopsies and*/*or sections of other extracutaneous organ(s) | - *>*25% of all MC are atypical type I or II cells on bone marrow sections/smears or are spindle-shaped in MC infiltrates detected in sections of bone marrow or other extracutaneous organs - Any *KIT* mutation with evidence of transforming behavior in the bone marrow or another extracutaneous organ - MC in bone marrow, blood, or another extracutaneous organ express CD2, CD25, and*/*or CD30 - Baseline serum tryptase *>*20 ng*/*mL, unless there is an unrelated myeloid neoplasm, in which case this parameter is not valid^a^ | 1 major +  1 minor criteria  *OR*  3 minor criteria^b^ |
| **Classification of Systemic Mastocytosis Subtype** | | |
| **B-findings**  **(“Burden of Disease”)** | **C-findings**  **(“Cytoreduction-Requiring”)** | **Diagnostic Requirements for Indolent Disease** |
| - High MC burden and neoplastic expansion (on BM biopsy): >30% infiltration of cellularity by MC and serum total tryptase >200 ng/mL - Signs of dysplasia or myeloproliferation in non-MC lineage(s), but criteria not met for definitive AHN diagnosis, with normal or only slightly abnormal blood counts - Hepatomegaly without impairment of liver function, palpable splenomegaly without hypersplenism, and/or lymphadenopathy on palpation or imaging^c^ - *KIT D816V* mutation with a variable allele frequency of ≥10% | - Bone marrow dysfunction caused by neoplastic MC infiltration, manifested by ≥1 cytopenia: - ANC < 1.0 × 10^9^ L^-1^ or - Hemoglobin <10 g/dL or - Platelet count <100,000 μL^-1^ - Palpable hepatomegaly with impairment of liver function, ascites, and/or portal hypertension^d^ - Skeletal involvement, with large osteolytic lesions^e^ with or without pathologic fractures - Palpable splenomegaly with hypersplenism^f^ - Malabsorption and/or life-threatening organ damage in other organ systems caused by local MC infiltration into tissues | **BMM:**  No skin lesions or B-findings;  Basal serum tryptase <125 ng/mL  **ISM:**   - No C-findings; - <2 B-findings   **SSM:**  No C-findings;   - ≥2 B-findings |
|  |  | **Diagnostic Requirements for Advanced Disease** |
|  |  | **SM-AHN:**   - Diagnostic criteria for MDS or MPN   **ASM:**   - ≥1 C-finding; - <20% MC in BM smears   **MCL:**  ≥20% MC in BM smear |

^a^If hereditary α-tryptasemia is also present, the tryptase level should be adjusted.

^b^Diagnostic requirements of International Consensus Classification of SM 2022 guidelines are 1 major criterion or 3 minor criteria (27).

^c^Organomegaly may be documented by palpation or imaging. Lymphadenopathy is defined as >2 cm by CT or ultrasound.

^d^Signs of impaired liver function include elevated transaminases and/or bilirubin levels and/or hyperalbuminemia (with or without ascites or portal tension).

^e^Large osteolytic lesions are defined as ≥2 cm.

^f^Hypersplenism is defined as splenomegaly in association with a cytopenia (eg, platelets <100,000 μL^-1^).

ANC, absolute neutrophil count; ASM, aggressive systemic mastocytosis; BM, bone marrow; BMM, bone marrow mastocytosis; CD, cluster of differentiation; ISM, indolent systemic mastocytosis; MC, mast cell; MCL, mast cell leukemia; MDS, myelodysplastic syndrome; MPN, myeloproliferative neoplasm; SM-AHN, systemic mastocytosis with an associated hematologic neoplasm; SSM, smoldering systemic mastocytosis.

Adapted from Valent et al. 2017^24^ and Khoury et al. 2022^25^

**References**

24. Valent P, Akin C, Metcalfe DD. Mastocytosis: 2016 updated WHO classification and novel emerging treatment concepts. Blood. 2017;129(11):1420-7.

25. Khoury JD, Solary E, Abla O, Akkari Y, Alaggio R, Apperley JF, et al. The 5th edition of the World Health Organization Classification of Haematolymphoid Tumours: Myeloid and Histiocytic/Dendritic Neoplasms. Leukemia. 2022;36(7):1703-19.
